# Characteristics of primary rat microglia isolated from mixed cultures using two different methods

**DOI:** 10.1186/s12974-017-0877-7

**Published:** 2017-05-08

**Authors:** Li Lin, Rakhi Desai, Xiaoying Wang, Eng H. Lo, Changhong Xing

**Affiliations:** 1 School of Pharmaceutical Sciences, Wenzhou Medical University, Wenzhou, Zhejiang 325035 China; 2 Neuroprotection Research Laboratory, Departments of Radiology and Neurology, Massachusetts General Hospital, Harvard Medical School, MGH East 149-2401, Charlestown, MA 02129 USA

**Keywords:** Microglia, Primary culture, Mild trypsinization, Shaking, Phenotype, Immune response

## Abstract

**Background:**

Microglial cultures comprise a critically important model system for investigating inflammatory mechanisms in almost all CNS disorders. Mild trypsinization and shaking are the two most commonly used methods to isolate primary microglia from mixed glial cultures. In this study, we characterized and compared microglia obtained using these two methods.

**Methods:**

Primary rat microglia cultures were prepared from cerebral cortices of 1–2-day-old neonatal Sprague-Dawley rats. After achieving confluency at about 14 days in vitro, microglia were isolated from mixed glial cultures via either mild trypsinization or shaking. The purity of microglia was estimated by flow cytometry. Quantitative real-time PCR was used to measure mRNA expression. TNFα, IL-1β, IL-10, and IGF-1 in cell culture supernatant were measured using ELISA kits. Phagocytic function was assessed using fluorescein-labeled *Escherichia coli* K-12 BioParticles.

**Results:**

Mild trypsinization generated a higher yield and purity than shaking. Microglia isolated by mild trypsinization appeared to be in a quiescent state with ramified morphology. Microglia isolated by shaking showed a more heterogenous morphology, including cells with rounded shapes suggestive of activation. Compared with shaking, microglia isolated by trypsinization also had lower baseline phenotype markers (iNOS, CD86, CD206, and arginase 1) and lower levels of cytokines (TNFα, IL-1β, IL-10, and IGF-1) as well as reduced phagocytic capability. Both methods yielded microglia that were responsive to various stimuli such as IL-4, lipopolysaccharide (LPS), or interferon-γ (IFNγ). Although stimulated patterns of gene expression and cytokine release were generally similar, there were also significant differences in terms of absolute response. LPS treatment induced significantly higher levels of TNFα and IL-10 in microglia isolated by mild trypsinization versus shaking. IFNγ induced a lower response in TNFα in microglia obtained by mild trypsinization versus shaking.

**Conclusions:**

Our results suggest that isolating microglia with the shaking method may induce slight activation even at baseline, and this may affect stimulus responses in subsequent experiments. Caution and attention should be warranted when choosing isolation protocols for primary microglia cultures.

## Background

Microglia are resident immune cells of the brain, constantly monitoring the microenvironment and responding to any kind of pathologic change. One striking feature of microglia is their highly dynamic nature [1]. Microglia can be activated by a large number of stimuli and change in their morphology, cytokine/chemokine expression profiles, and function. Depending on the stimulus and context, activated microglia exhibit a spectrum of phenotypic and functional diversity, ranging from the so-called classically activated (M1-like) to alternatively activated (M2a, M2b, and M2c) [2–9]. M1-like microglia promote the release of various proinflammatory cytokines, thus inducing bystander tissue injury [9–15]. By contrast, M2-like macrophages may actively promote tissue remodeling and repair [9, 12–17].

Primary microglia cultures comprise a useful in vitro tool for exploring a wide range of inflammatory mechanisms in central nervous system (CNS) disease and investigating therapeutic strategies that may target microglia. In vitro, M1-like phenotype is achieved by exposing cells to lipopolysaccharides (LPS) and interferon-γ (IFNγ), whereas interleukin (IL)-4 and IL-13 are commonly used to induce M2-like phenotype [18]. However, there are many ways to prepare primary microglia, and some caution may be warranted because these highly reactive cells may respond differently under different isolation conditions. In this study, we isolated microglia from rat brains using two commonly used methods (shaking versus mild trypsinization) and assessed and compared their morphology, gene expression profiles, and cytokine release under baseline and stimulated conditions.

## Methods

### Reagents

DMEM/F12, 0.25% trypsin-EDTA, and fetal bovine serum (FBS) for cell culture were from Gibco.

Primary antibody against Iba-1 and Alexa Fluor 555-conjugated secondary antibody were from Abcam and Molecular Probe, respectively. The antibodies of CD11b, CD45, and CD68 for flow cytometry detection were purchased from BD, eBioscience, and Bio-Rad, respectively, and were diluted according to the manufacturer’s instructions. Recombinant rat IL-4 was from Amsbio. LPS was from Sigma-Aldrich. Recombinant rat IFNγ was from R&D. The stock solutions of IL-4, LPS, and IFNγ were prepared using sterile ddH_2_O, and ddH_2_O was used as control.

### Primary rat microglia culture

**Fig. 1:**
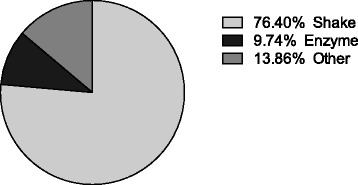
The proportion of various methods that are used for primary rodent microglial culture by searching PubMed using the term “microglia AND (rat OR mouse) AND primary AND culture NOT review [publication type]”

A PubMed search was conducted using the terms “microglia AND (rat OR mouse) AND primary AND culture NOT review [publication type]” from 2006 to 2016. This search resulted in 392 articles. One hundred twenty-five articles were excluded (81 did not use microglial culture, 25 microglia used cell lines, 19 were not available for full-text access), resulting in 267 articles that used primary cultures of rodent microglia. This quick survey of the literature suggested that two most commonly used methods to purify microglia involved shaking and mild trypsinization (204 used shaking and 26 of them used mild trypsinization) (Fig. 1). Based on this initial analysis, we compared microglia obtained with shaking versus trypsinization. Primary rat microglia cultures were prepared from cerebral cortices of 1–2-day-old neonatal Sprague-Dawley rats. After removing the meninges, the cortical tissues were digested with 0.25% trypsin-EDTA for 30 min at 37 °C, followed by mechanical triturating in DMEM/F12 with 10% fetal bovine serum. The mixed cortical cells were passed through a 70-μm nylon mesh cell strainer and plated on non-coated plastic dishes or plates in DMEM/F12 with 10% FBS, and the medium was completely replaced every 3–4 days. After achieving confluency at about 14 days in vitro, the microglia were isolated from mixed glial cultures via either mild trypsinization (enzyme, E) or shaking (S). The mild trypsinization was performed according to previously described methods [19, 20]. Incubation of mixed glial cultures with a trypsin solution (0.25% trypsin-EDTA diluted 1:4 in DMEM/F12) for 15–25 min resulted in the detachment of an intact layer of cells in one piece. Microglial cells remained attached to the bottom of the well. For the shaking method [21, 22], confluent mixed glial cultures were placed on an orbital shaker at 220 rpm for 1 h. The supernatant containing the detached microglial cells was collected and re-seeded for 1 h to allow microglial attachment. After 1 h, the nonadherent cells were removed. Microglia isolated from both methods were allowed to rest overnight prior to treatments. To compare the yield, we plated the cells from one brain of neonatal pups into one 6-well plate. Yield was calculated as cell numbers per field (six random fields of ×200 magnification per culture, *n* = 5 cultures). The cells were fixed in 4% paraformaldehyde for 30 min, blocked with 5% normal horse serum for 1 h, and incubated with primary antibody against Iba-1 (1:100) at 4 °C overnight. After washing, the cells were incubated with Alexa Fluor 555-conjugated secondary antibodies (1:200) for 1 h at room temperature. Negative controls were incubated without primary antibodies, and no immunoreactivity was observed in these controls.

### Flow cytometry

The purity of microglia obtained from mild trypsinization or shaking was estimated by flow cytometry. The cells were collected and labeled with fluorochrome-conjugated monoclonal antibodies recognizing antigens (CD11b, CD45, and CD68) at 4 °C for 15 min. After labeling, the cells were washed twice in PBS and resuspended at a final volume of 400 μl. Flow cytometry was performed on a BD LSDII, and data were analyzed using FlowJo software.

### Real-time PCR

Quantitative real-time PCR was used to measure mRNA expression. Cultured microglia were treated with IL-4 (20 ng/ml), LPS (50 ng/ml), or IFNγ (20 ng/ml) for 8 h. Total RNAs were extracted using miRNeasy kit (Qiagen) from primary cultured microglia with or without treatment. One hundred nanograms of total RNAs were reverse transcribed into cDNA using M-MLV reverse transcriptase (Invitrogen). Quantitative expression of inducible nitric oxide synthase (iNOS), CD86, CD206, arginase 1, CX3C chemokine receptor 1 (CX3CR1), toll-like receptor 2 (TLR2), and C-C chemokine receptor 2 (CCR2) were measured using gene-specific TaqMan Gene Expression Assays (ABI 7500HT, Applied Biosystems). Relative baseline gene levels were calculated by subtracting Ct value of β2-microglobulin (B2M) from Ct value of detected genes. Changes in gene expression (fold change) after various treatments were determined using the 2^−ΔΔCt^ method with normalization to B2M. All real-time PCRs were performed in triplicates. All experiments were repeated three to six times independently. Activated microglia include a spectrum of various states. The representative genes we selected, including iNOS, CD86, CD206, arginase 1, CX3CR1, TLR2, and CCR2, are closely relevant to different activating states of microglia. To compare the “average fingerprint” of cultured microglia, quantitative data were analyzed using two-way ANOVA (*p* < 0.05 for significance, SPSS 21).

### ELISA

Cultured microglia were treated with IL-4 (20 ng/ml), LPS (50 ng/ml), or IFNγ (20 ng/ml) for 24 h. Tumor necrosis factor α (TNFα) (eBioscience), IL-1β (R&D), IL-10 (eBioscience), and insulin-like growth factor-1 (IGF-1) (R&D) in cell culture supernatant were measured using ELISA kits, according to the manufacturer’s instructions.

### Microglia phagocytic function assays

To assess phagocytosis, microglial cells cultured in 6-well plates were digested using 0.25% trypsin-EDTA and re-seeded to a 96-well plate at a concentration of 3.0 × 10^4^ cells/well. Then, the cells were incubated with fluorescein-labeled *Escherichia coli* K-12 BioParticles (Invitrogen) for 2 h at 37 °C. The cells were rinsed with 0.25 mg/ml trypan blue to quench extracellular fluorescence. Intracellular fluorescence was read using a fluorescence microplate reader setup with excitation at 480 nm and emission at 520 nm. The experiments were performed with five replicates per condition and repeated four times.

### Statistical analysis

Data were expressed as mean ± SE. Three to six separate experiments were performed. Data of real-time PCR were analyzed using two-way ANOVA. Data of ELISA that measures the cytokine release after various treatments were analyzed using one-way ANOVA. Other data were analyzed using *t* test between two isolation methods. Statistical significance was set at *p* < 0.05.

## Results

### Morphology, yield, and purity

**Fig. 2:**
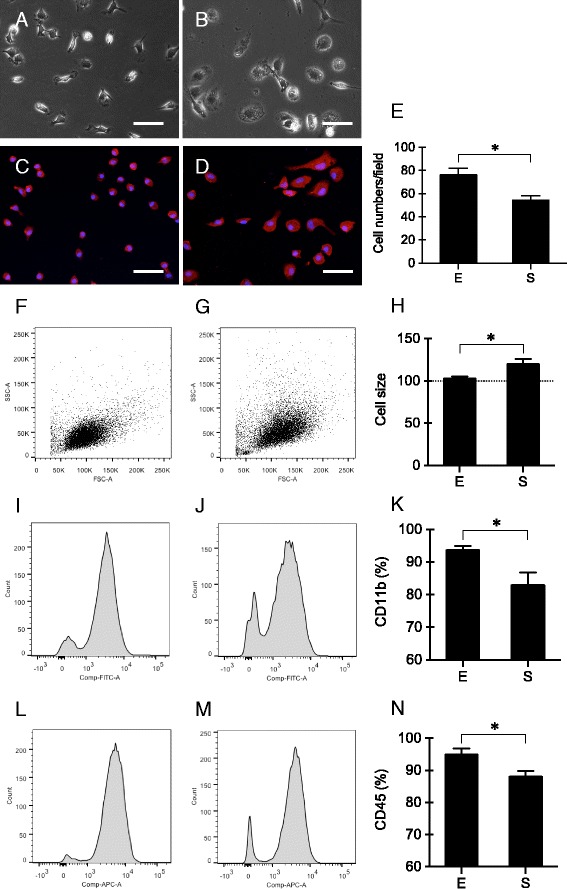
Morphology, yield, and purity of primary cultured microglia obtained using mild trypsinization (*E*) or shaking (*S*). **a**, **b** Morphology of primary microglia isolated using mild trypsinization (**a**) or shaking (**b**). *Scale bar* = 50 μm. **c**, **d** Iba-1 immunostaining of primary microglia isolated using mild trypsinization (**c**) or shaking (**d**). *Scale bar* = 50 μm. **e** The yield of microglia cultures. *n* = 5. **f**–**h** Size of primary microglia isolated using mild trypsinization (**f**) or shaking (**g**). *n* = 5. **i**–**n** Purity of primary microglia isolated using mild trypsinization (**i**, **l**) or shaking (**j**, **m**) was determined by flow cytometry with CD11b (**i**–**k**) or CD45 (**l**–**n**) staining. *n* = 5. *p < 0.05

Phase contrast microscopy revealed distinct morphology of microglia isolated using two different methods. Microglia isolated using mild trypsinization showed uniform ramified morphology with short processes and small cell body (Fig. 2a). The morphology of microglia isolated using shaking was more heterogeneous. Most of them showed enlarged round cell body with reduced processes (Fig. 2b). Cells from both methods were Iba-1 positive (Fig. 2c, d). The yield of microglia cultures was higher by using mild trypsinization (76.18 ± 13.08 cells/field) than by using shaking (54.27 ± 9.19 cells/field) (*p* = 0.015, *t* test) (Fig. 2e).

Besides morphology, the size of microglial cells obtained from the two methods was also different. Measured by flow cytometry with forward scatter (FSC), the average size of microglia isolated using shaking was 1.17-fold larger than those obtained using mild trypsinization (*p* = 0.030, *t* test) (Fig. 2f–h). The purity of cultured microglia was determined by flow cytometry with CD11b and CD45 staining. The proportion of CD11b-positive cells (Fig. 2i–k) and CD45-positive cells (Fig. 2l–n) were significantly higher in the mild trypsinization group (93.68 ± 2.54% and 94.92 ± 3.64%) compared to the shaking group (82.9 ± 7.61% and 88.08 ± 3.32%) (*p* = 0.028 for CD11b, *p* = 0.024 for CD45, *t* test).

### Baseline gene expression, cytokine production, and phagocytic function

**Fig. 3:**
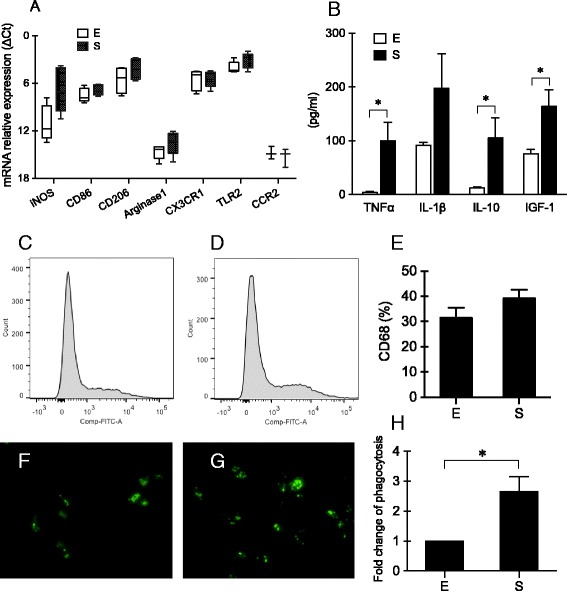
Baseline levels of gene expression, cytokine release, and phagocytosis of primary cultured microglia isolated using mild trypsinization (*E*) or shaking (*S*). **a** Baseline levels of gene expression. *n* = 3–6. **b** Baseline levels of TNFα, IL-1β, IL-10, and IGF-1 in microglial-conditioned media. *n* = 4. **c**–**e** Flow cytometry showed CD68-positive cells of primary microglia isolated using mild trypsinization (**c**) or shaking (**d**). *n* = 5. **f**–**h** Phagocytosis of primary microglia isolated using mild trypsinization (**f**) or shaking (**g**). *n* = 4. *p < 0.05

Because the morphology of microglia appeared to be different in the two preparation methods, we next asked whether these cells might also demonstrate different phenotypes. A representative panel of genes was examined to assess various activation states—iNOS, CD86, CD206, arginase 1, CX3CR1, TLR2, and CCR2. Baseline expression of these selected genes were significantly different in microglia obtained by shaking versus mild trypsinization (*p* = 0.003, 0.000, and 0.036 for methods, genes, and methods*genes, respectively, two-way ANOVA) (Fig. 3a).

Microglia are known to influence adjacent cells via the release of extracellular signals. Therefore, we used ELISA to assess key cytokines (TNFα, IL-1β, and IL-10) and the major microglial growth factor IGF-1 (Fig. 3b). Baseline levels of IL-1β (*p* = 0.122, *t* test) and IGF-1 (*p* = 0.032, *t* test) were about 2-fold higher in conditioned media from cultured microglia isolated by shaking compared with mild trypsinization. Even larger differences were observed for IL-10 (9-fold, *p* = 0.048, *t* test) and TNFα (24-fold, *p* = 0.020, *t* test), again with significantly higher levels from microglia isolated using shaking than using mild trypsinization.

In general, these differences in gene expression and cytokine release suggested that microglia isolated with shaking may be “more activated” compared with those obtained via mild trypsinization. CD68 is considered to be a general marker of activated microglia. Flow cytometry showed that the percentage of CD68-positive cells was slightly higher in microglia isolated using shaking (39.14 ± 6.94%) than using mild trypsinization (31.58 ± 7.80%), but there was no significant difference between the two methods (*p* = 0.185, *t* test) (Fig. 3c–e). However, an in vitro assay demonstrated that phagocytic capacity was significantly enhanced by about 2.5-fold in microglia by shaking versus mild trypsinization (*p* = 0.018, *t* test) (Fig. 3f–h).

### Comparative response to stimulation

**Fig. 4:**
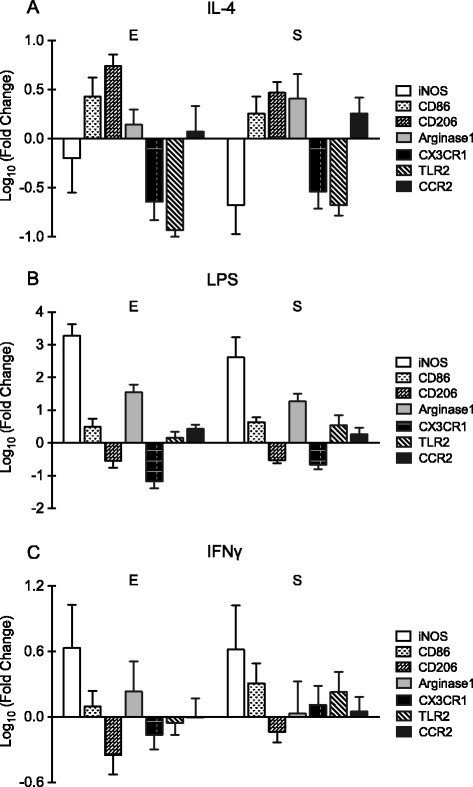
Gene expression changes of primary cultured microglia isolated using mild trypsinization (*E*) or shaking (*S*) after treatment with 20 ng/ml of IL-4 (**a**), 50 ng/ml of LPS (**b**), or 20 ng/ml of IFNγ (**c**) for 8 h. *n* = 3–6

**Fig. 5:**
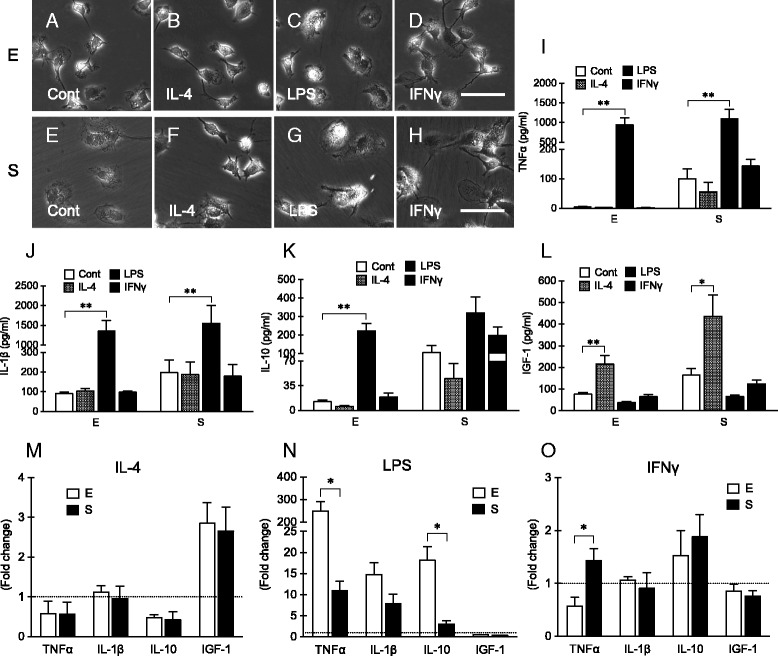
Morphological change (**a**–**h**) and cytokines release (**i**–**l**) of primary cultured microglia isolated using mild trypsinization (*E*) or shaking (*S*) after treatment with 20 ng/ml of IL-4, 50 ng/ml of LPS, or 20 ng/ml of IFNγ for 24 h. *n* = 4. *Scale bar* = 50 μm. **m**–**o** Reanalysis of data of cytokines release from primary cultured microglia isolated using mild trypsinization (*E*) or shaking (*S*) after treatment with 20 ng/ml of IL-4 (**m**), 50 ng/ml of LPS (**n**), or 20 ng/ml of IFNγ (**o**) for 24 h. Data were presented as fold changes compared with control. *p < 0.05; **p < 0.01

Next, we tested the response of microglia isolated by these two methods to three typical stimuli that were commonly used to activate microglia into different phenotypes, i.e., IL-4, LPS, and IFNγ. After treatment with IL-4 (Fig. 4a) or LPS (Fig. 4b) for 8 h, the pattern of gene expression response (iNOS, CD86, CD206, arginase 1, CX3CR1, TLR2, and CCR2) appeared to be generally similar (for IL-4 treatment, *p* = 0.881, 0.000, and 0.408 for methods, genes, and methods*genes, respectively; for LPS treatment, *p* = 0.962, 0.000, and 0.430 for methods, genes, and methods*genes, respectively, two-way ANOVA). For example, both IL-4 and LPS treatment decreased CX3CR1 expression. IL-4 treatment upregulated the expression of M2-like phenotype marker CD206, whereas LPS treatment upregulated the expression of M1-like phenotype marker iNOS and downregulated CD206. The responses to 8-h IFNγ treatments were slightly more variable but once again, overall patterns were similar (*p* = 0.333, 0.023, and 0.916 for methods, genes, and methods*genes, respectively, two-way ANOVA). IFNγ increased the expression level of iNOS and decreased the level of CD206 in microglia from both shaking and trypsinization groups (Fig. 4c). Consistent with these gene expression findings, morphological changes also appeared to be mostly the same in both groups after various treatments for 24 h (20 ng/ml of IL-4, 50 ng/ml of LPS, or 20 ng/ml of IFNγ) (Fig. 5a–h).

For further comparisons, the conditioned media after 24 h treatment with the IL-4, LPS, or IFNγ stimuli were collected to measure the levels of secreted cytokines and growth factors. Consistent with the baseline data described above, pre-stimulation levels of TNFα, IL-1β, IL-10, and IGF-1 were significantly higher in conditioned media of microglia isolated using shaking compared with trypsinization. Stimulation with IL-4, LPS, or IFNγ induced distinct responses (Fig. 5i–l, one-way ANOVA). LPS significantly affected TNFα, IL-1β, and IL-10. IL-4 significantly increased the production of IGF-1, but it had no effect on IL-10, TNFα, and IL-1β. IFNγ had little effects. The direction of the response (increase or decrease) was mostly similar in microglia isolated with both methods (Fig. 5i–l). For example, in microglia isolated from either mild trypsinization or shaking, IL-4 treatment increased IGF-1 release, and LPS treatment increased the generation of TNFα and IL-1β. However, there were some key differences in the extent of response in some cases. To assess differences of the extent of response, we recalculated the data as fold change versus control in Fig. 5m–o. Compared with the baseline release, LPS treatment induced significantly higher levels of TNFα and IL-10 in microglia obtained by mild trypsinization than by shaking (Fig. 5n). For IFNγ stimulation, TNFα release was significantly elevated in microglia isolated using shaking, but not in microglia isolated using mild trypsinization (Fig. 5o).

## Discussion

Microglia serve as critical sensors, effectors, and regulators for CNS homeostasis during development and in health and disease [23–25]. Even in healthy brain, microglia are not functionally silent cells. They are highly active, extending and retracting motile processes through which they survey their microenvironment and interact dynamically with surrounding cells [24, 26–28]. Accumulating knowledge now suggest that microglia have multifactorial effects far beyond their traditional roles in immunity [29]. Microglia remove apoptotic neurons, both during CNS development and in adult brains [30–34]. Microglia modulate neurogenesis and promote wiring during embryogenesis and adulthood [35–38]. Microglia take part in synaptic pruning and remodeling in the brain [39–42]. Any insult to the CNS, including infection, trauma, or metabolic dysfunction, causes microglial activation. Upon activation, microglia undergo morphological and functional changes [2, 43]. Microglia can produce numerous mediators including cytokines (both proinflammatory and anti-inflammatory), chemokines, growth factors, and neurotrophins. Microglia can also be phagocytic and generate reactive oxygen and nitrogen species. Therefore, investigations into microglial mechanisms are critically important for a wide spectrum of neuroscience.

Primary cultures comprise a vital in vitro tool for studying microglia. A number of protocols were available for culturing primary microglial cells from neonatal rodent brains. Historically, rodent microglia are isolated using the shaking method [21, 22]. In 2003, another method was introduced that involved mild trypsinization [19]. Although some new methods have been demonstrated to achieve high-yield isolation of microglial cells from postnatal and adult brains [44, 45], shaking remains the most commonly used approach to date (see Fig. 1). Because microglia are highly sensitive and reactive cells, it is possible that different preparation protocols may result in slightly different phenotypes of microglial cells, which in turn can potentially influence experimental outcomes. Therefore, the purpose of the present study was to directly compare key features of microglia isolated using these two methods. Compared to the shaking method, mild trypsinization method to separate microglia from mixed glial cultures generated a higher yield as well as purity. More important is that the shaking method appeared slightly activate microglial cells even under normal condition. Microglial activation is not univalent or bivalent. The concept of classically activated (M1-like) to alternatively activated (M2-like) is derived from the studies of macrophages. Microglia differ from macrophages that reside in other tissues based on their cell-specific gene expression signatures, distinct ontogeny, and differential functions [23, 46–48]. Depending on the stimulus and context, activated microglia exhibit a wide spectrum of phenotypic and functional diversity, not only M1- or M2-like. The so-called M1- or M2-like markers are also not always absolutely limited to one microglial phenotype. Microglia isolated by shaking showed “ameboid” morphology. Compared with mild trypsinization, the baseline expression of microglial activation markers (iNOS, CD86, CD206, and arginase 1) and the baseline release of cytokines (TNFα, IL-1β, IL-10, and IGF-1) were significantly higher in microglia isolated by shaking. Microglia isolated using shaking also showed enhanced phagocytosis when compared with the microglia isolated using mild trypsinization. These findings further confirm that primary cultured microglia are extremely sensitive to stimuli, and responses are sometimes dependent on isolation protocols. Hence, caution may be required in choosing methods and interpreting data. For studies that focus on baseline microglial physiology, it may be better to select methods that minimize inadvertent activation. Caveats must also be acknowledged when investigating pathways by probing with a recombinant protein, especially if the purity is lower and the level of endotoxin in the recombinant protein is higher.

In our study, we documented the changes of gene expression (iNOS, CD86, CD206, arginase 1, CX3CR1, TLR2, and CCR2) and cytokine release (TNFα, IL-1β, IL-10, and IGF-1) in microglia obtained from these two methods in response to various typical stimuli that may induce M1-like (LPS or IFNγ) or M2-like (IL-4) phenotypes. The microglia obtained by either mild trypsinization or shaking were fully functional and generally responded to different treatments as expected. However, the extent of responses appeared to be different under some conditions. Even though both methods were feasible for evaluating functional responses of microglia in vitro, opposite conclusions may arise if the extent of response was critical for a particular study. For example, to evaluate the beneficial or harmful effects of activated microglia, the levels of TNFα released from activated microglia may be a key. Different concentrations of TNFα offer distinct receptor selectivity [49]. Higher levels of TNFα may induce neuronal death and increase glutamate release, but lower levels of TNFα may not [50, 51]. In this scenario, the different responses of TNFα to LPS or IL-4 stimulation in microglia isolated by shaking or trypsinization may affect results and conclusions.

There are a few caveats. First, the purity of cultured cells is one of the critical factors that would influence the experimental results. Unfortunately, it is almost impossible to obtain 100% pure cells in any primary culture. In our primary microglial cultures, other types of brain cells, including neurons, astrocytes, and oligodendrocytes, might exist. However, the differences in purity might not be a major contributor to the differential inflammatory responses or baseline immune mediator expression in this study because the genes and cytokines we measured are mainly expressed by microglia, rather than neurons, astrocytes, or oligodendrocytes. Actually, one potential advantage of using mild trypsinization to culture microglia may be because this method provides about 95% pure microglial cells. Nevertheless, if these cultures were further passaged, other cells may still persist and the proportion of various cell types might be changed. This is an important caveat. Second, the speed and duration of shaking might be important to influence the characteristics of microglia isolated by shaking. Shaking speed and duration to isolate microglia from mixed cultures are variable in the literatures. In the present study, we used shaking speed and duration that were typically used in our lab. Compared with the literature, 220 rpm is a commonly used speed [52, 53]. Third, since microglia are very sensitive to even tiny stimulations, it is possible that the phenotype of microglia would be switched if the microglia were allowed to recuperate for a longer time after being isolated from the mixed cultures, such as 48 h instead of overnight. Future studies to assess various time points after the shaking method would be beneficial. Fourth, although we showed that the yield was higher using the mild trypsinization method than the shaking method in the present study, we acknowledge that we do not know what causes the difference of the yield in the two methods. We plated cells from one neonatal brain into one 6-well plate to make sure that the initial culture conditions are same in both methods. Before taking the photos to compare the yield, we cultured the microglial cells for another 24 h after isolating them from the mixed cultures using either shaking or mild trypsinization. However, potential differences in microglial mechanisms of response to enzyme versus mechanical manipulations remain to be elucidated.

Recently, other studies have also raised the possibility that the shaking method might inadvertently activate microglia, and immunomagnetic microbead methods have been proposed as an alternative way for in vitro isolation [52, 54, 55]. Here, we showed that the mild trypsinization method resulted in similar purity as the magnetic microbead method. The purity of mild trypsinization method used in the present study is about 95%. The purity of magnetic microbead method is about 95–97% according to the literature [52, 54, 55]. Compared with magnetic sorting, mild trypsinization might also be cheaper (no need for extra reagents and equipment) and simpler (no extra procedures).

## Conclusions

In summary, mild trypsinization may be a reliable method to isolate microglia from mixed glial cultures with increased yield and purity, and microglial purified by mild trypsinization may be closer to their “resting” state. The immune state of microglia was influenced by the method of purification, and the culture method should be carefully considered in in vitro research.

## Abbreviations

B2M: β2-Microglobulin
CCR2: C-C chemokine receptor 2
CNS: Central nervous system
CX3CR1: CX3C chemokine receptor 1
E: Mild trypsinization
FBS: Fetal bovine serum
IFNγ: Interferon-γ
IGF-1: Insulin-like growth factor-1
IL: Interleukin
iNOS: Inducible nitric oxide synthase
LPS: Lipopolysaccharides
S: Shaking
TLR2: Toll-like receptor 2
TNFα: Tumor necrosis factor α
